# Quinic acid derivatives inhibit dengue virus replication in vitro

**DOI:** 10.1186/s12985-015-0443-9

**Published:** 2015-12-22

**Authors:** Paula Rodrigues Zanello, Andrea Cristine Koishi, Celso de Oliveira Rezende Júnior, Larissa Albuquerque Oliveira, Adriane Antonia Pereira, Mauro Vieira de Almeida, Claudia Nunes Duarte dos Santos, Juliano Bordignon

**Affiliations:** Laboratório de Virologia Molecular, Instituto Carlos Chagas, ICC/Fiocruz, 81350-010 Curitiba, PR Brazil; Departamento de Química, Universidade Federal de Juiz de Fora, 36036-330 Juiz de Fora, MG Brazil

**Keywords:** Dengue, Replication, Antivirals, Quinic acid

## Abstract

**Background:**

Dengue is the most prevalent arboviral disease in tropical and sub-tropical areas of the world. The incidence of infection is estimated to be 390 million cases and 25,000 deaths per year. Despite these numbers, neither a specific treatment nor a preventive vaccine is available to protect people living in areas of high risk.

**Results:**

With the aim of seeking a treatment that can mitigate dengue infection, we demonstrated that the quinic acid derivatives known as compound **2** and compound **10** were effective against all four dengue virus serotypes and safe for use in a human hepatoma cell line (Huh7.5). Both compounds were non-virucidal to dengue virus particles and did not interfere with early steps of the dengue virus life cycle, including binding and internalization. Experiments using a replicon system demonstrated that compounds **2** and **10** impaired dengue virus replication in Huh7.5 cells. Additionally, the anti-dengue virus effects of the quinic acid derivatives were preserved in human peripheral blood mononuclear cells.

**Conclusions:**

Taken together, these data suggest that quinic acid derivatives represent a novel chemical class of active compounds that could be used to combat dengue virus infection.

**Electronic supplementary material:**

The online version of this article (doi:10.1186/s12985-015-0443-9) contains supplementary material, which is available to authorized users.

## Background

Among all human arthropod-borne viral diseases, dengue is the most prevalent, representing a health threat in tropical and sub-tropical areas of the world [1, 2]. There are approximately 2.5 billion people living in endemic areas, and 390 million dengue cases are estimated per year including 25,000 dengue-related deaths [1, 3].

Dengue virus (DENV) belongs to the *Flavivirus* genus (*Flaviviridae* family) and comprises four distinct serotypes: DENV-1, −2, −3 and −4. The virus is transmitted to humans by female *Aedes* spp. mosquitoes during their blood meals [2, 4]. The DENV serotypes are genetically distinct despite having a similar epidemiology, and they are all able to cause the same disease in humans [5, 6]. Each ~50 nm viral particle is surrounded by a lipid bilayer that is derived from the host cell. The single-stranded positive RNA genome is approximately 10.7 kb in length and presents a single open reading frame (ORF) [5, 6] that encodes three structural proteins that are related to particle formation: C (capsid), pre-M/M (membrane and its precursor) and E (envelope). It also encodes seven non-structural proteins (NS) that are involved in RNA replication and immune evasion: NS1, NS2A, NS2B, NS3, NS4A, NS4B and NS5 [5–9].

After a prodromal period of 4–10 days, patients who are infected with dengue will either remain asymptomatic or present with the following clinical forms: (i) dengue without warning signs (vomiting, rash, achiness, leucopenia, positive tourniquet test), (ii) dengue with warning signs (abdominal pain, persistent vomiting, fluid accumulation, mucosal bleeding, lethargy, liver enlargement, increasing hematocrit with decreasing platelets) or (iii) severe dengue (SD; severe plasma leakage, severe bleeding, or organ failure) [1].

It is noteworthy that despite the vast number of dengue cases that have been identified and despite their severity, to date neither a specific dengue treatment nor an approved vaccine to prevent infection has been developed. Hence, the recognition of dengue signs and the local epidemiological conditions that are associated with medical care are important for reducing the mortality that is associated with the disease [1, 10]. The development of a specific dengue therapy has been challenging. Each structure/protein that is involved in the viral life cycle can serve as a target for the development of novel antiviral agents, and the use of compound libraries appear to be the most effective strategy in searching for active compounds against flaviviruses [11].

Quinic acid (Table 1) is a carboxylated cyclohexanepolyol that is found in several vegetables (potato, carrot, tomato, coffee) and exists either in free form or as esters [12]. It is widely used as an optically-active synthetic precursor in multistep chemical synthesis [13], and it is the starting material that is used for the synthesis of Tamiflu, a drug used in the treatment of influenza A and B [14]. Additionally, quinic acid derivatives are found in propolis produced by *Apis mellifera* (European honey bee) in the south and southeast regions of Brazil [15]. Furthermore, it has been shown that quinic acid derivatives possess antiviral activities against Human Immunodeficiency Virus (HIV) [16–18], Hepatitis B Virus (HBV) [17, 19], and Herpes Simplex Virus 1 (HSV-1) [20, 21].

**Table 1:**
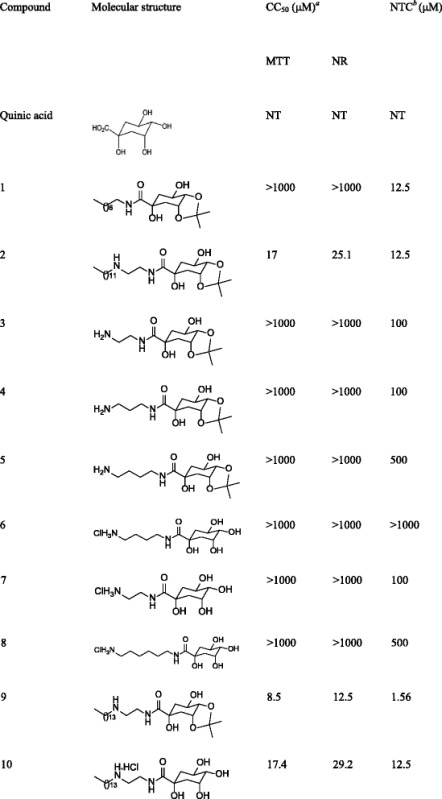
Molecular structures of quinic acid derivatives and cytotoxicity evaluations in Huh7.5 cells

^*a*^CC50: cytotoxic concentration in 50 % of cells

^*b*^NTC: non-toxic concentration in Huh7.5 cells

NT: not-tested

In this study, we demonstrated that the amides of quinic acid derivatives present anti-dengue virus activity in vitro in Huh7.5 cells and human PBMCs. Furthermore, we revealed that quinic acid derivatives impair dengue virus replication in Huh7.5 cells.

## Results and discussion

### Cytotoxicity of quinic acid derivatives

Both quinic acid (Table 1) and several of its derivatives have been shown to protect human lymphocytes from damage induced by X-ray [22] and from cell death induced by tetrahydropapaverolin [23]. Table 1 shows the quinic acid derivatives that were tested in the present study. Recently, it has been demonstrated that the amides of quinic acid derivatives exhibited anti-inflammatory activities both in vitro and *in vivo* and therefore they may serve as attractive options for therapeutic use [24, 25]. Furthermore, one of these amides was found to enhance the survival of C57/Bl6 mice that were exposed to lethal radiation by 45 % [26]. Additionally, a quinic acid ester (QAE) prolonged cell survival by reducing replication in S-phase cells, indicating that it protects cells from damage by allowing time for cellular DNA damage repair to occur [27].

In addition to their individual protective potentials, the safe concentrations of each compound were determined in Huh7.5 cells. To accomplish this, the in vitro toxicities of the quinic acid derivatives were determined by MTT, which is a tetrazolium salt that is metabolized by cellular reductases only in cells with viable mitochondrial activity [28]. An assessment of Neutral Red (NR) uptake, which demonstrates a cell’s ability to incorporate red dye into lysosomes that maintain physiological pH [29], was performed simultaneously with the MTT assay in the same cell cultures [30]. Based on the results from both assays, it was possible to determine a non-toxic concentration (NTC) of each compound for Huh7.5 as well as the cytotoxic concentration for 50 % of the culture (CC_50_; Table 1). The data show that quinic acid derivatives presented a wide range of cytotoxicity in Huh7.5 cells, with CC_50_ values varying between 1.56 and >1000 μM.

### Antiviral activity

The quinic acid derivative 3,5-dicaffeoyl-muco-quinic acid has been shown to impair HIV integrase activity and inhibit viral replication in vitro [31]. Quinic, chlorogenic and caffeic acids exhibited anti-HBV activity in vitro in HepG2.2.15 cells. Crude extracts of regular and decaffeinated coffee also inhibited HBV replication [19]. Additionally, 3,5-dicaffeoylquinic acid exhibited specific activity against respiratory syncytial virus (RSV). However, this compound was not able to inhibit influenza A and B subtypes or herpes simplex 1 and 2 [32].

**Fig. 1:**
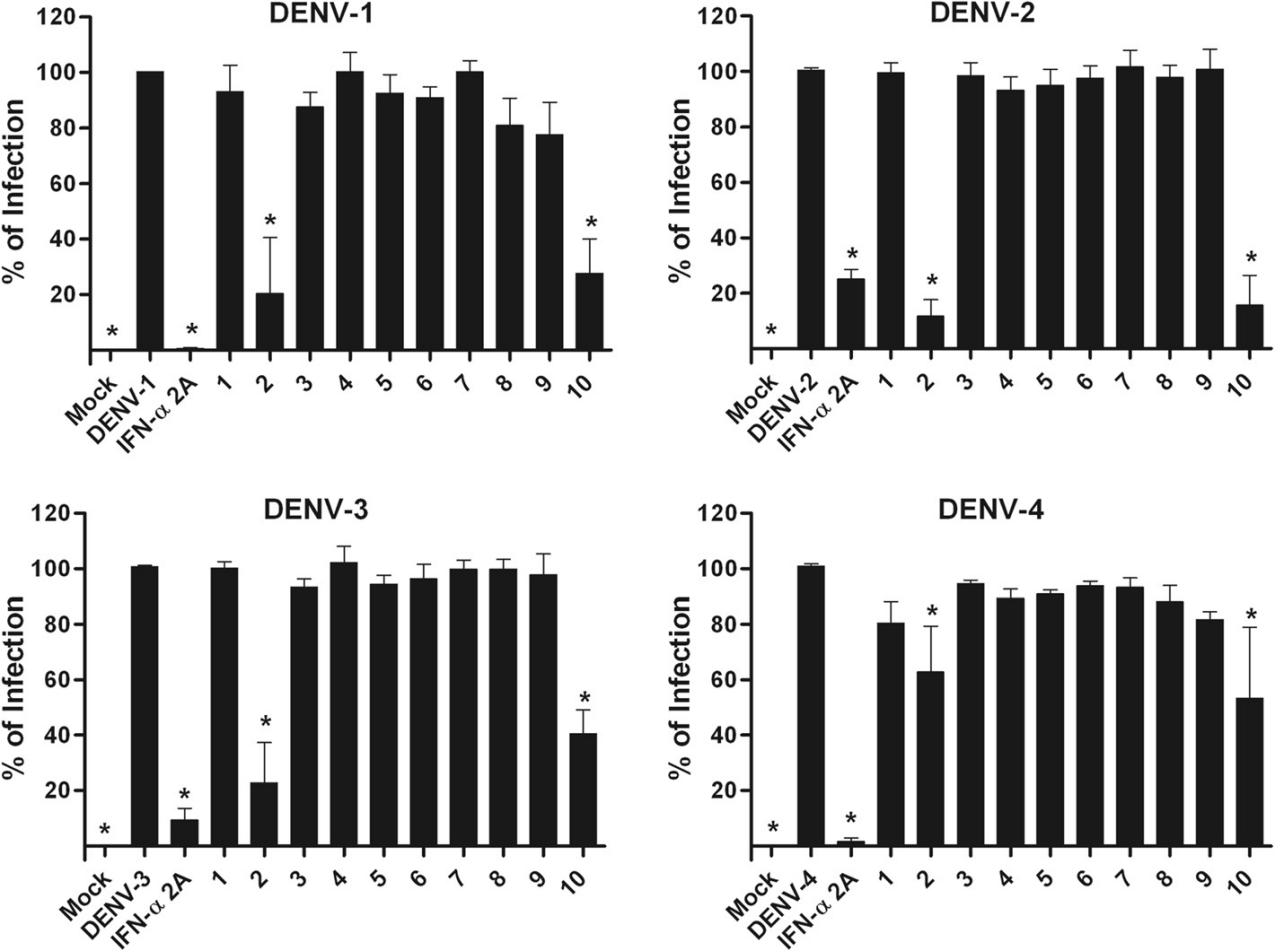
Antiviral screening. Huh7.5 cells were infected and treated during and after infection. After 72 h, the results from the *in situ* ELISA assay indicated the most promising substances (*p < 0.05 compared to DENV control). Data represent the mean ± standard error (SE) from three independent experiments (*p < 0.05 compared to DENV control)

**Fig. 2:**
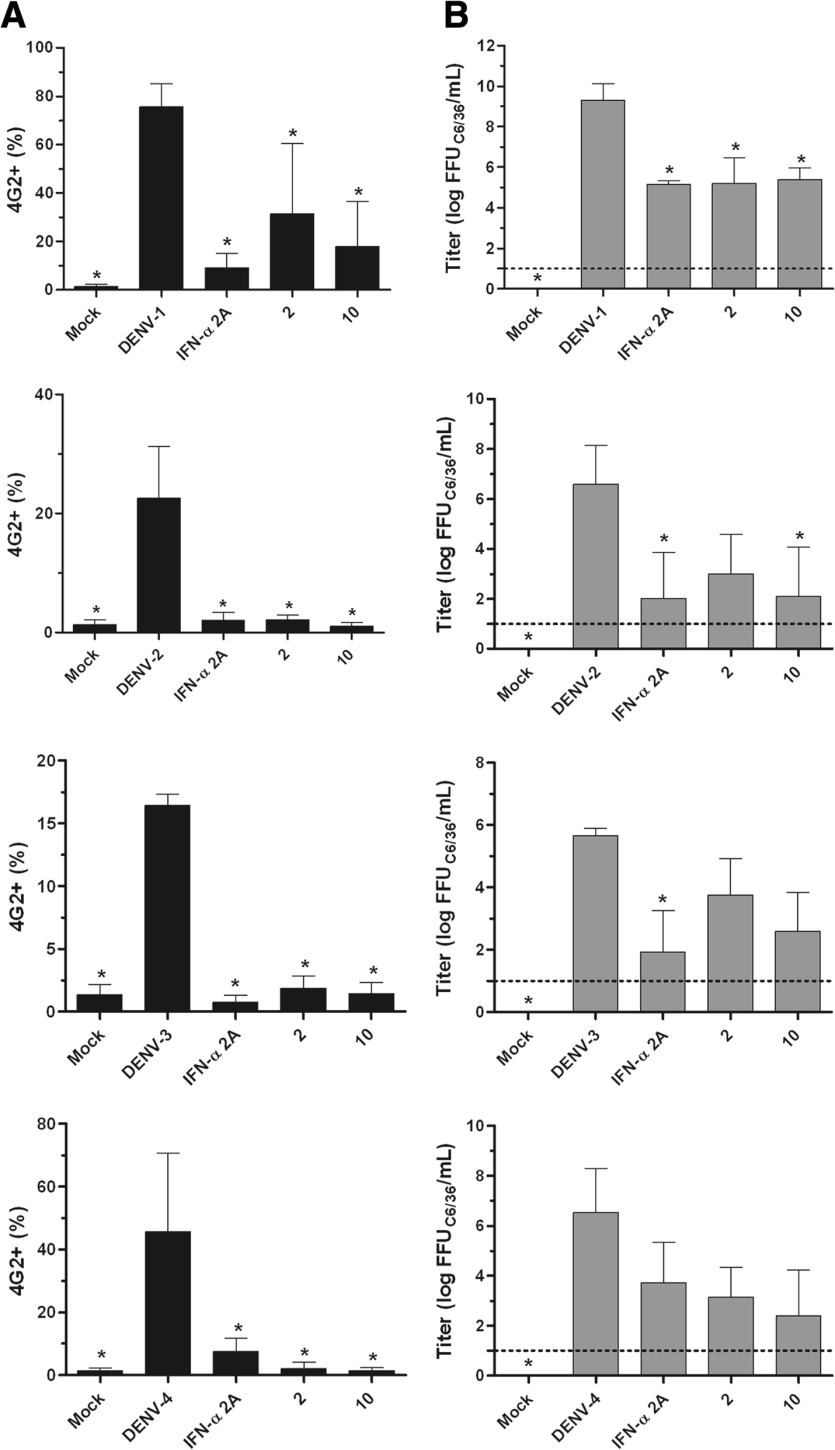
Antiviral activity confirmation by secondary assays. Huh7.5 cells were infected with DENV-1 through −4 and treated during and after infection with each substance (NTC). After 72 h, the cells were submitted to FACS (**a**), and supernatants were used in focus-forming assays in C6/36 cells (**b**). Data represent the mean ± standard error (SE) from three independent experiments (*p < 0.05 compared to DENV control)

It is notable that, to the best of our knowledge, the current study is the first to evaluate the activity of quinic acid derivatives against flavivirus replication. When screened in Huh7.5 cells using an *in situ* ELISA assay [33], two of the quinic acid derivatives, called compounds **2** and **10,** that were included in this study demonstrated anti-dengue virus activity at varying levels by reducing cell infectivity for all four dengue serotypes (Figs. 1 and 2). These data suggest that the presence of a lipophilic chain could contribute for the observed antiviral activity, as the compounds that did not possess this moiety were either less active or not active.

To confirm the antiviral activity of compounds **2** and **10**, a flow cytometry assay was employed [34]. Data from FACS analysis confirmed that these compounds were able to reduce the percentage of cells infected with DENV (Fig. 2a). Furthermore, a titration of culture supernatants led to a notable reduction in virus titers (Fig. 2b), which corroborated the previous results.

The concentration-response curves starting from the NTCs of compounds **2** and **10** showed different SIs for each dengue virus serotype (Table 2 and Additional file 1: Figure S1).

**Table 2:** Concentrations that inhibit 50 % of infection and the selective indexes of compounds **2** and **10** with respect to the four dengue virus serotypes

	2		10	
	IC_50_ ± SD (μM)	SI	IC_50_ ± SD (μM)	SI
DENV-1	6.9 ± 3.6	3.6	6.5 ± 0.2	4.5
DENV-2	10.4 ± 3.9	2.4	8.7 ± 2.3	3.3
DENV-3	10.3 ± 3.1	2.4	10.3 ± 3.8	2.8
DENV-4	9.23 ± 3.7	2.7	10.8 ± 1.7	2.7

### Dengue virus replication is impaired by compounds 2 and 10

**Fig. 3:**
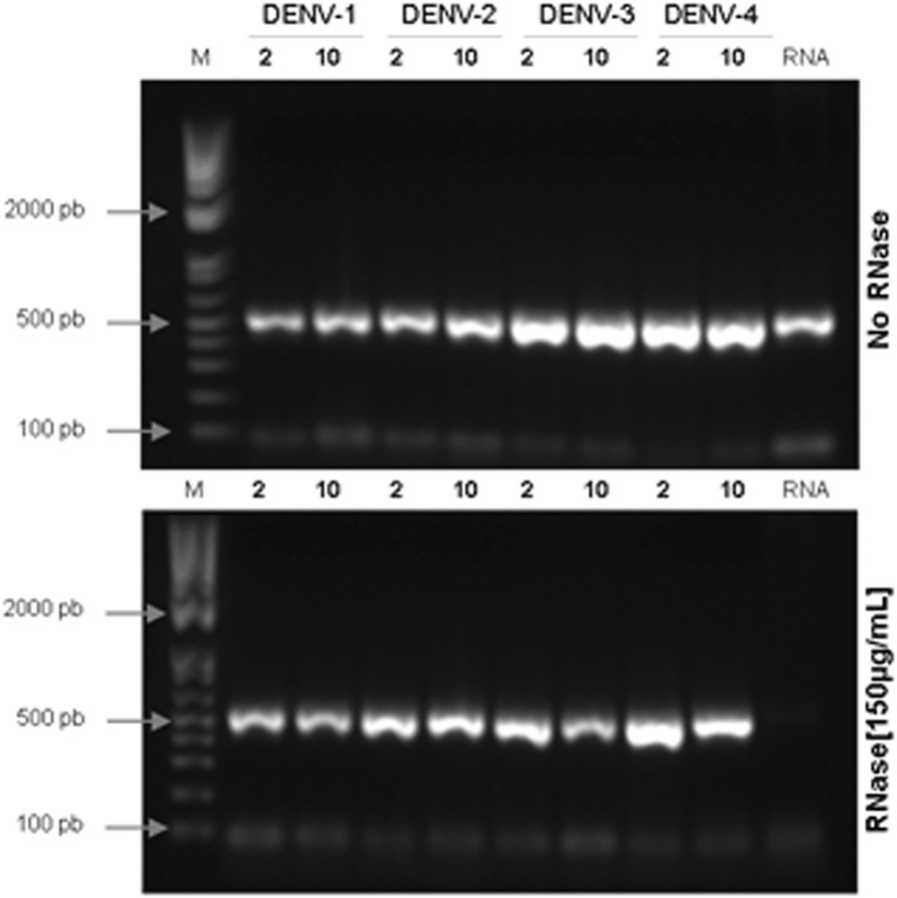
Virucidal activity. DENV serotypes 1 through 4 were incubated with compounds **2** and **10** (NTC) in the presence or absence of RNase. RNA samples were extracted and submitted to RT-PCR and gel electrophoresis. Representative data from 4 independent assays. M: 1 kb DNA ladder; RNA: viral RNA control; bp: base pairs

After confirming the antiviral activity of compounds **2** and **10**, we aimed to define which stage(s) of the viral infection cycle was being affected by these compounds. First, virucidal effects were assessed as previously described [35], as it has been demonstrated that dicaffeoylquinic acid exhibited a virucidal effect against RSV at high concentrations that was dependent on temperature [36]. The results indicated that the compounds did not destroy viral particles, as demonstrated by the amplification of RNA after RNase treatment (Fig. 3).

**Fig. 4:**
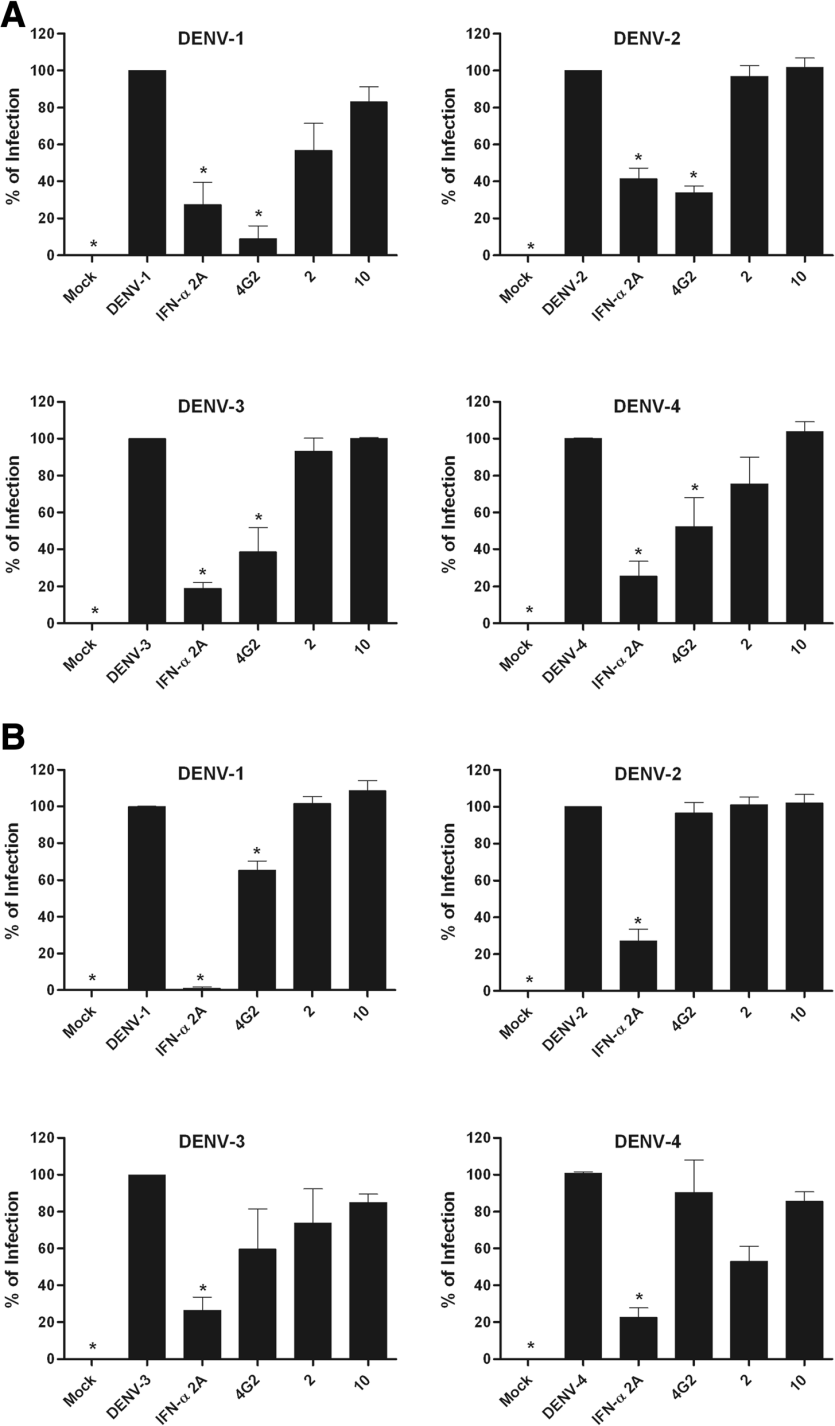
Adsorption and internalization assays. Binding inhibition assay (**a**) and inhibition of virus internalization (**b**) for each DENV serotype. After 72 h incubation period the *in situ* ELISA was performed. Data represents mean ± standard error of 3 independent assays (*p < 0.05 compared to DENV control)

Furthermore, we evaluated whether the compounds could affect the early steps of viral infection in host cells. The treatment of cells with compounds **2** and **10** was performed during virus binding and internalization in independent assays. The results demonstrated that neither compounds reduced the percentage of infected cells in comparison to controls (Fig. 4). There, it could be suggested that they interfere with other steps in the dengue virus life cycle. Li et al., [36] showed that dicaffeoylquinic acid does not affect viral attachment to host cells; however, it did inhibit virus-cell fusion during the early stages of viral infection, as well as cell-to-cell fusion during the final step of the RSV replication cycle.

Our next goal was to evaluate whether compounds **2** and **10** would affect later steps of the dengue virus life cycle, such as viral replication. To perform this assay, a replicon system for dengue virus serotypes −1 and −3 (RepDV1 and RepDV3, respectively) was used [37, 38]. In this system, a subgenomic RNA contains the non-structural viral proteins that are required for RNA replication and translation but does not contain the structural proteins and therefore viral particles are not assembled. Employing RNA replicons enables the study of antivirals that specifically inhibit steps during viral replication and/or translation. Ng et al., [39] had developed a Renilla luciferase-reporter dengue virus type 2 replicon and a stable BHK-21 cell harboring the replicon and used them to test nucleoside inhibitors of NS5 and siRNA against NS3.

**Fig. 5:**
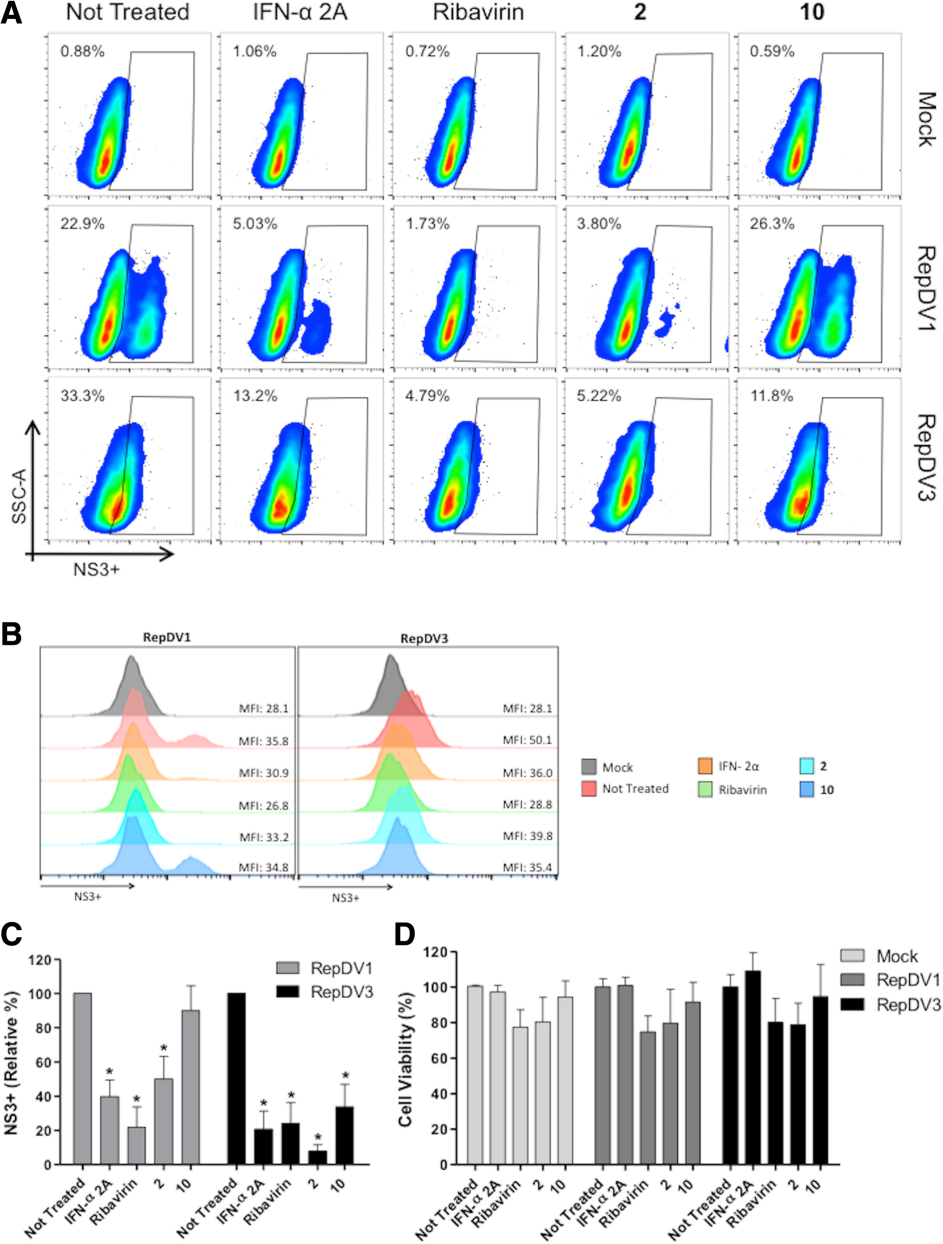
DENV subgenomic replicon system. Huh7.5 cells were transfected with either dengue virus serotype-1 replicon RepDV1 or dengue virus serotype-3 replicon RepDV3 RNA, and after one hour elapsed they were treated with compounds **2** and **10** at the NTC. After 72 h, the cells were submitted to FACS analysis (anti-NS3 staining using the monoclonal antibody 1722). Pseudocolor plots (**a**) and histograms showing the mean fluorescent intensity (MFI) (**b**) are representative of one experiment, and relative percentages as mean ± standard error (SE) from three independent experiments (**c**). In parallel, cell viability was evaluated by neutral red assay (**d**). *p < 0.05 compared to the untreated control

The results showed that we succeeded in transfecting Huh7.5 cell cultures with both replicons and that the concentrations of compounds **2** and **10** that were employed in this assay were non-toxic to Huh7.5 cells. Figure 5 illustrates that compound **2** acted on dengue virus replication (RepDV1 and RepDV3). Furthermore compound **10** inhibited dengue virus replication only for serotype-3 (RepDV3), not showing any effect against replication of dengue virus serotype-1 (RepDV1). Data suggests that for dengue virus serotype-1 another target in the late stages of the viral life cycle (such as viral assembly and/or release) could be inhibited. A serotype dependence in anti-dengue virus activity has been previously demonstrated for sulfated polysaccharides from marine seaweeds [40] and for kinase-binding-site compounds [41]. Additionally, dicaffeoylquinic acid derivatives have been shown to impair HIV-1 replication in infected cells by disturbing anti-HIV-1 integrase activity [18]. Also, chlorogenic, quinic and caffeic acid were able to inhibit HBV-DNA replication in HepG2.2.15 [19].

### Anti-dengue virus activity in human peripheral blood mononuclear cells

After demonstrating that the antiviral activity of the tested compounds occurs during DENV replication in Huh7.5 cells, it was necessary to evaluate the effects of compounds **2** and **10** in human PBMCs to simulate a more physiologically relevant situation. The NTCs of compounds **2** and **10** in human PBMCs were assessed before performing antiviral assays. After treating PBMCs with varying concentrations of each compound, cells were incubated for 5 days and then stained with annexin V and propidium iodide to detect apoptosis and/or cell death. The compounds were more toxic to human PBMCs than to Huh7.5 cells (data not shown).

**Fig. 6:**
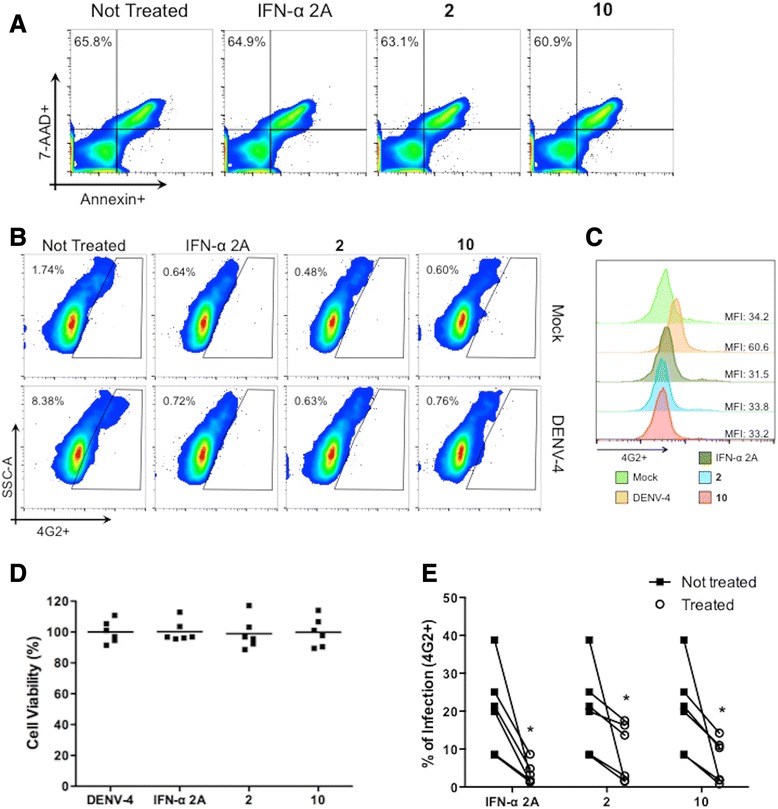
Antiviral activity in PBMCs. PBMCs were infected with DENV-4 (MOI 10) and treated with each compound. After 5 days in culture, cells were assayed for apoptosis (**a, d**) and infection (**b, c** and **e**) by flow cytometry. The figure includes the pseudocolor plot data from one representative blood donor and the mean from 6 healthy donors. *p < 0.05 compared to the untreated control

After setting a non-toxic dose of 3.12 μM for PBMCs, cells from six healthy human donors were infected with DENV-4, as a proof of concept, and treated with compounds **2** and **10** both during and after the infection. DENV-4 (strain TVP360) was selected once it was the most resistant tested-serotype to compounds **2** and **10** treatment (see Fig. 1). After 5 days in culture, the compounds were demonstrated to be safe, and they exhibited efficacy against dengue virus serotype 4 infection (Fig. 6).

## Conclusions

This study demonstrated that two different derivative amides of quinic acid were effective against all four dengue virus serotypes when used in Huh7.5 cells in vitro. Also it was shown that the two compounds are safe for Huh7.5 cells and PBMCs. Importantly, the results from experiments that were performed using a replicon system suggested that compounds **2** and **10** inhibited dengue virus replication. Both compounds were also effective against dengue virus infection in human PBMCs. To our knowledge, this is the first description of anti-dengue virus activity in quinic acid derivatives. These findings offer a new perspective for the development of anti-dengue virus therapy based on quinic acid derivatives. Of note, there is currently no approved antiviral treatment for dengue disease. We are currently synthesizing novel derivatives in an attempt to improve antiviral activity and to further examine structure-activity relationships to improve SI for compounds **2** and **10**.

## Methods

### Cell lines and viruses

*Aedes albopictus* mosquito cells C6/36 (ATCC: CLR-1660) were maintained at 27 °C in Leibovitz’s Medium (L-15; Gibco-Invitrogen, USA) that was supplemented with 0.26 % tryptose (Sigma-Aldrich, USA), 5 % Fetal Calf Serum (FCS; Gibco-Invitrogen, South America) and 25 μg/mL gentamicin (Gibco-Invitrogen, China).

Huh7.5 human hepatoma cells (PTA-8561, U.S. Patent Number 7455969) were grown in Dulbecco’s Modified Eagle Medium - nutrient mixture F-12 (DMEM-F12, Gibco-Life Technologies, USA) that was supplemented with 100 IU/μg/mL penicillin/streptomycin (Gibco-Invitrogen, USA) and 10 % FCS. Upon project approval by the FIOCRUZ Committee of Ethics in Research (#514/09), primary Peripheral Blood Mononuclear Cells (PBMCs) were isolated from whole blood samples taken from healthy volunteers with lymphocyte separation medium (Lonza, USA) by density gradient centrifugation, in accordance with the manufacturer’s recommendations. PBMCs were cultured in 24-well plates with Roswell Park Memorial Institute medium-1640 (RPMI-1640, Lonza, USA) that was supplemented with 2 mM glutamine (Gibco-Life Technologies, USA), 100 IU/μg/mL penicillin/streptomycin, 2.5 μg/mL amphotericin B (Cristália, Brazil), 100 mM sodium pyruvate (Sigma-Aldrich, USA) and 10 % FCS. Both Huh7.5 cells and PBMCs were maintained in a humid 37 °C atmosphere with 5 % CO_2_.

DENV-1/FGA/89 was isolated in 1989 from a South American patient suffering from dengue fever (GenBank: AF226687). DENV-2/ICC-265 and DENV-3/97 are clinical isolates from Brazilian patients who had dengue fever in 2009 and 2004, respectively. DENV-4/TVP360 is a laboratory strain that was kindly provided by Dr. Ricardo Galler (Fundação Oswaldo Cruz, Rio de Janeiro, Brazil). Viruses were grown in insect C6/36 cells, and culture supernatants were titrated using a focus immunodetection assay [42].

### Synthesis

Ten (**1**–**10**) amides of quinic acid derivatives were synthesized as previously described [43]. Compounds were prepared both with and without lipophilic chains to investigate the influence of lipophilicity on antiviral activity (Table 1). All compounds were recovered in 100 % dimethyl sulfoxide (DMSO, Sigma-Aldrich, USA) and stored at −20 °C. The maximum concentration of DMSO that was used in cell culture assays was 0.5 %. There were no difference in control treatments with or without 0.5 % DMSO.

### Cytotoxicity assays

Huh7.5 cells were treated with the compounds ranging from 1000 to 0.5 μM, and cell viability was measured after 72 h simultaneously by MTT [3-(4,5-Dimethylthiazol-2-yl)-2,5-diphenyltetrazolium bromide] and Neutral Red (NR) assays, as previously described [30]. Data from three independent experiments were normalized with the following equation: cell viability (%) = (OD sample value - OD blank control)/(OD cell control - OD blank control) × 100. The non-toxic concentration (NTC) of each compound was determined using both assays and defined as the highest concentration that did not show significant differences from the non-treated control (one-way ANOVA and Dunnett’s post-test). CC_50_ was calculated using a sigmoidal dose response curve (variable slope).

To establish NTCs in PBMCs, serial dilutions of compounds **2** and **10** were tested after 5 days of treatment, using Annexin V-PE-Cy7 and 7-AAD (Apoptosis Detection Kit, Becton & Dickinson, EUA) according to the manufacturer’s instructions and were analyzed by flow cytometry using a BD FACS Canto II (Flow Cytometry Facility RPT08L PDTIS/Carlos Chagas Institute - Fiocruz, PR-Brazil).

### Antiviral screening of compounds

The antiviral activities of ten quinic acid derivatives were screened using *in situ* ELISA [33]. Briefly, Huh7.5 cells (2x10^4^ cells/well in 96-well plates) were infected with DENV-1, −2 and −3 with a MOI of 4 and DENV-4 with a MOI of 0.1. The NTCs of the compounds were used to treat cells both during and after infection (to cover all steps of the virus life cycle). After 72 h, cells were fixed with methanol:acetone for 1 h at −20 °C, blocked with 2 % skim milk and 0.05 % Tween-20 in PBS for 30 min, and then incubated with the 4G2 mouse monoclonal antibody that is specific to flavivirus envelope protein for 1 h at 37 °C. Following this, cells were washed four times with washing buffer (0.01 % Tween 20 in PBS) and a secondary goat anti-mouse IgG HRP antibody (Sigma-Aldrich, USA) was added. After 1 h incubation at 37 °C, cells were washed four times, and TMB substrate (KPL, USA) was added for 10 min under protection from light. The reaction was stopped with the addition of 2 M H_2_SO_4_. Absorbance was read at a wavelength of 450 nm in a microplate reader (Synergy H1M, Biotek, USA). Data were normalized as % of infection compared to controls; non-infected cells (mock) were considered to represent 0 % infection, and untreated infected cells were considered to represent 100 % infection. Recombinant IFN-α-2A (100 IU/mL) was used as a reference control, and compounds were considered as active when 70 % of inhibition of at least one serotype was achieved.

Furthermore, concentration response curves were obtained using serial dilutions of the active compounds, starting from their NTCs. The concentration that inhibited 50 % of virus infection (IC_50_) was obtained using nonlinear regression followed by sigmoidal concentration-response (variable slope; GraphPad) and selectivity index (SI = CC_50_/IC_50_).

### Antiviral activity confirmation by supplementary assays

The active compounds that were obtained from the initial screening were confirmed by two methods. Huh7.5 cells were infected with DENV-1 through −4 and treated during virus inoculation. Following this, media that contained compounds **2** or **10** was added to the cells and incubated with them for 72 h. After the incubation period, cell culture supernatants were recovered to perform a foci-forming immunodetection assay in C6/36 cells, as previously described [42]. Huh7.5 cells were recovered, blocked for 20 min at room temperature (PBS, 5 % FCS), fixed with Cytofix/Cytoperm™ (BD Biosciences) and stained with the anti-Flavivirus 4G2 mouse monoclonal antibody in Perm/Wash solution (BD Biosciences) for 20 min at 37 °C. After washing with Perm/Wash, the cells were stained with rabbit anti-mouse IgG (H + L) Alexa-633 (Life Technologies) for 20 min at 37 °C. Finally, cells were washed two times with 1x PBS and analyzed using a BD FACS Canto II (BD Biosciences).

### Virucidal assay

A virucidal assay was performed as previously described [35] with minor modifications. Briefly, samples of each DENV serotype (2x10^5^ ffu/mL) were treated with the NTCs of the active compounds (**2** and **10**) in the presence or absence of 150 μg/mL RNase A (USB-Affymetrix Inc.) for 1 h at 37 °C. After treatment, viral RNA was extracted using a QIAamp Viral RNA Mini Kit (QIAGEN). The RNA was reverse-transcribed using 250 pmol of a random primer (Invitrogen, USA) and *Improm II Reverse Transcriptase* (Promega, USA). Amplification by PCR was performed as described by Lanciotti et al. [44] with some modifications. Briefly, cDNA was amplified using D1 (5′- TCAATATGCTGAAACGCGCGAGAAACCG - 3′) and D2 primers (5′- ATTGCACCAGCAGTCAACGTCATCTGGTTC - 3′) with Taq DNA polymerase. Samples were maintained at 94 °C for 3 min, followed by 35 cycles of 94 °C for 30 s, 55 °C for 30 s and 72 °C for 1 min in a GeneAmp PCR System 9700 (Applied Biosystems, USA). Recently extracted DENV-3/97 RNA samples, that were either treated or not treated with RNase, were used as the positive and negative controls, respectively.

### Viral binding and internalization assays

To perform a binding assay, Huh7.5 cells were seeded in 96-well plates (2x10^4^ cells/well), infected with DENV-1, −2, −3 (MOI 4) and −4 (MOI 0.1) and treated with the active compounds. After 1 h at 4 °C, cells were washed twice with cold PBS, and the viral inoculum was replaced with complete media. After incubation for 72 h at 37 °C and 5 % CO_2_, the *in situ* ELISA was performed.

An internalization assay was performed by infecting Huh7.5 cells as described above for 1 h at 4 °C. Following this, cells were washed twice with cold PBS, and the active compounds were added. After another hour of incubation at 37 °C, the cells were washed and treated with citrate buffer (citric acid 40 mM, potassium chloride 10 mM, sodium chloride 135 mM, pH 3.0) for 1 min to remove non-internalized viral particles. After washing, cells received complete media and were incubated at 37 °C, 5 % CO_2_ for 72 h until analysis by *in situ* ELISA. The mouse monoclonal 4G2 antibody (neutralizing antibody against flavivirus) and recombinant IFN-α 2A (100 IU/mL) were used as controls for both assays.

### Transient replicon assay

To quantify the inhibition of RNA replication by the active compounds, two transient replicon assays were used: RepDV1 generated from dengue virus serotype-1 BR/90 strain (GenBank AF226685) and RepDV3 generated from dengue virus serotype-3 BR DEN3 290–02 strain (GenBank EF629369) [37, 38]. DNA plasmids were purified from the *Escherichia coli* Top10 strain using a Wizard Plus Midiprep DNA Purification System (Promega, Madison, WI, USA) following manufacturer’s recommendations. Plasmids were linearized with the SwaI restriction enzyme and submitted to phenol/chloroform extraction and ethanol precipitation. An in vitro transcription reaction using DNA templates was achieved using a MEGAscript T7 High Yield Transcription Kit (Ambion, Austin, TX, USA) in the presence of an m^7^G(5′)ppp(5′) RNA Cap analog (New England Biolabs, Ipswich, MA, USA). RNA purification was performed with an RNeasy kit (QIAGEN, Valencia, CA, USA). Finally, the resulting RNAs were used to transfect Huh7.5 cells (2 ng RNA/ 2x10^6^ cells) following the recommendations of the manufacturer of the Amaxa Cell Line Nucleofector Kit T and Nucleofector II/2B device (Lonza, Cologne, Germany).

After transfection, cells were plated in 24- (1x10^5^ cells) and 96-well (2x10^4^ cells) plates. Treatments with the NTCs of the active compounds were performed one hour after transfection, and the plates were incubated for an additional 72 h. After this period, the cells from the 24-well plates were recovered, blocked for 20 min at room temperature (PBS, 5 % FCS), fixed with Cytofix/Cytoperm (Becton & Dickinson, San Jose, CA) and stained with the 1722 mouse monoclonal antibody (anti-NS3 recombinant protein from dengue virus serotype-1) in Perm/Wash solution (Becton & Dickinson, San Jose, CA) for 20 min at 37 °C. The mouse monoclonal antibody 1722 recognizes dengue virus serotypes 1, 2 and 3. (data not shown). After washing with Perm/Wash, cells were stained with anti-mouse Alexa-633 (Life Technologies) for 20 min at 37 °C. Finally, cells were washed two times with Perm/Wash and analyzed using a BD FACS Canto II (Becton & Dickinson, San Jose, CA). The cells in the 96-well plates were submitted to a cell viability neutral red assay [29].

Non-RNA-transfected and non-treated cells were used as mock controls. Huh7.5 cells that were transfected with RNA and that were not treated were used as a positive control for virus replication. Cells that were treated with recombinant IFN-α 2A (100 IU/mL) and 20 μM ribavirin were used as reference controls.

### Antiviral effect in primary human cells

Peripheral blood mononuclear cells (PBMCs) were infected with DENV-4 (MOI 10) for 2 h and treated with the NTCs of the active compounds for five days at 37 °C and 5 % CO_2_. After incubation, cells were analyzed for DENV antigen quantification by FACS. Briefly, the cells were blocked with PBS, 5 % FCS (Gibco-Invitrogen, South America) and 1 % human serum type AB (Lonza, Walkersville, MD) for 20 min at room temperature. Following this, the cells were fixed using Cytofix/Cytoperm (Becton & Dickinson, San Jose, CA), washed using Perm/Wash, and stained with the 4G2 monoclonal (specific for flavivirus envelope protein) for 20 min at 37 °C. After incubation, the cells were washed with Perm/Wash and incubated for 20 min at 37 °C with the secondary antibody (donkey anti-mouse conjugated with Alexa-488; Life Technologies). Finally, cells were washed twice with Perm/Wash and analyzed using a FACSCanto II (BD Biosciences). Data were analyzed by two-way ANOVA followed by the Bonferroni post test.

### Data analysis

Statistical analyses were performed using Prism software (GraphPad version 5.0, USA), with a significance of *p* < 0.05. Flow cytometry data were analyzed by FlowJo version X software (Tree Star Inc., USA).

## Additional file

Additional file 1: Figure S1. Concentration response curve for compounds **2** and **10** in Huh7.5 cells infected with all four dengue virus serotypes. Cells were infected with DENV and treated during and after the infection in a range of concentrations. Mean ± SE of three independent experiments. IC_50_ was calculated using a sigmoidal dose response curve (variable slope). (TIF 607 kb)
