# Supplementary figures and images for: Quinic acid derivatives inhibit dengue virus replication in vitro
Source: Virol J. 2015 Dec 22;12:223. doi: 10.1186/s12985-015-0443-9 (PMC4688969; doi:10.1186/s12985-015-0443-9)

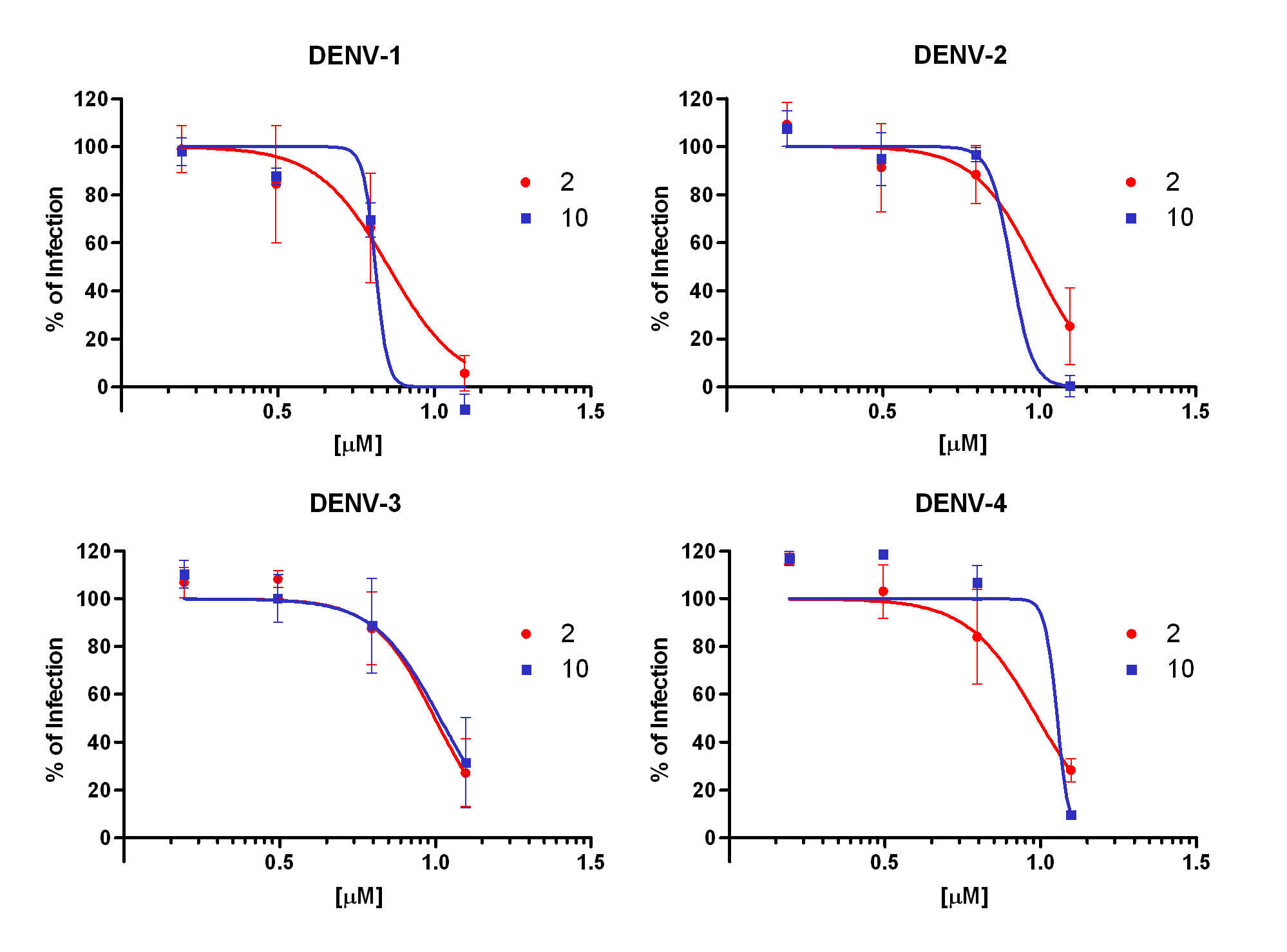

Supplement: Additional file 1: Figure S1. — Concentration response curve for compounds 2 and 10 in Huh7.5 cells infected with all four dengue virus serotypes. Cells were infected with DENV and treated during and after the infection in a range of concentrations. Mean ± SE of three independent experiments. IC50 was calculated using a sigmoidal dose response curve (variable slope). (TIF 607 kb) [file 12985_2015_443_MOESM1_ESM.tif]
